# Patient safety culture and associated factors among health care professionals at public hospitals in Dessie town, north east Ethiopia, 2019

**DOI:** 10.1371/journal.pone.0245966

**Published:** 2021-02-04

**Authors:** Fentaw Mohammed, Mekuanint Taddele, Tenaw Gualu

**Affiliations:** 1 Department of Nursing, Dessie Health Science College, Dessie, Amhara, Ethiopia; 2 Department of Public Health, College of Health Sciences, Debre Markos University, Amhara, Ethiopia; 3 Faculty of Medicine, The Nethersole School of Nursing, The Chinese University of Hong Kong, New Territories, Hong Kong, Hong Kong; United Nations Population Fund, BANGLADESH

## Abstract

**Introduction:**

Patient safety culture is defined as the attitudes, perceptions, and values that staffs share within an organization related to patient safety. The safety of health care is now a major global concern. It is likely that millions of people suffer disabling injuries or death directly related to medical care. Particularly in developing and transitional countries, patient harm is a global public health problem. The objective of the study is to assess patient safety culture and associated factors among health care professionals working in public hospitals in Dessie town, North East Ethiopia, 2019.

**Methods:**

Facility based quantitative study was employed from March 15 –April 30, 2019 in public hospitals in Dessie town. Four hundred and twenty two health care professionals were recruited to complete a structured pretested self-administered questionnaire. The data was cleaned, coded and entered in to Epi Info-7 and exported to SPSS version 20. Data was further analyzed using bivariate and multivariate logistic regression analyses. Variables with P value of less than 0.05 in multivariate analysis were declared as statistically significant at 95% CI.

**Results:**

Of the 422 recruited a total of 411 participants completed the survey with a response rate of 97.4%. Close to half (184(44.8%)) of the participants indicated good patient safety culture. Good patient safety culture was positively associated with working in primary hospital (AOR = 2.56, 95% CI = 1.56, 4.21). On the other hand, good patient safety culture was negatively associated with health professional’s age between 25–34 year (AOR = 0.25, 95% CI = 0.08–0.74) and working in Pediatrics ward (AOR = 0.39, 95% CI = 0.17–0.9) and in emergency ward (AOR = O.25, 95%CI = 0.09–0.67).

**Conclusion:**

The overall level of patient safety culture was under 50%. Good patient safety culture had positive association with working in primary hospital and negative association with professionals’ age between 25–29 year, 30–34 year and working in pediatrics and emergency ward. Implementing actions that support all dimensions of safety culture should be promoted at all levels of hospitals.

## Introduction

Patient safety is the absence of preventable harm to a patient during the process of health care and reduction of risk of unnecessary harm associated with health care to an acceptable minimum [1]. An acceptable minimum refers to the collective notions of given current knowledge, resources available and the context in which care was delivered weighed against the risk of non-treatment or other treatment [1].

Unsafe medical care is responsible for an enormous human toll [2]. Approximately 134 million adverse events occur each year in hospitals in low and middle income countries that contribute to 2.6 million deaths annually due to unsafe care [1]. In an Eastern Mediterranean and African study almost one third of patients who suffered a harmful incident died, 14% sustained permanent disability, 16% sustained moderate disability, 30% were left with minimal disability and 8% of the patients’ harm could not be specified [2]. In low and middle income countries(LMICs), a combination of numerous factors such as understaffing, inadequate organizational structures, overcrowding, lack of health care commodities, a shortage of basic equipment, and poor hygiene and sanitation contribute to unsafe patient care [3].

Patient safety is now being recognized as a large growing global public health challenge. Global efforts to reduce the burden of patient harm have not achieved substantial change over the past 15 years despite pioneering work in some health care settings [4]. However, there have been limited systemic improvements in the safety of health care globally, and in some situations efforts made have been sustained and uncoordinated [5]. Even in LMICs these measures have not been successfully adapted and applied [4].

Although new knowledge is required to measure and understand the risks and causes of harm and to develop solutions that prevent, reduce or mitigate the effects of harm, patient safety research is still in its infancy [6]. Particularly, investigation of patient safety in developing countries has been infrequent and limited in scope [7]. Now, there is growing concern for making systems safe and developing patient safety culture. One way to identify the existing gaps is through research and there is limited research being conducted in Ethiopia.

As a result, conducting a study on patient safety culture was one way to identify the current problem. Therefore, this study can be used as a reference for health care providers, health care educators, policy makers, and future researchers.

## Methods

### Study area and period

The study was carried out in Dessie town, Amhara region state of Ethiopia from March 15 –April 30/2019.

### Study design

Facility based quantitative study was employed.

### Study population

All health care professionals who were working at public hospitals in Dessie town who fulfil the inclusion criteria

### Inclusion criteria

All health care professionals who were working at public hospitals in Dessie town for at least one year.

### Sample size

The sample size was determined by using single population proportion formula and considering (46.7%) prevalence of positive patient safety from research done previously in Jimma zone hospitals (95% confidence interval, 5% marginal error, 10% response rate) [8]. Hence, the total sample size calculated was 422.

### Recruitment and sampling procedure

This study was conducted in Dessie referral hospital and Boru Meda Primary hospital. There were a total of 538 and 222 health care professionals in Dessie referral hospital and Boru Meda primary hospital respectively. The study participants were proportionally allocated for each hospital based on the total number of health care professionals in each discipline. Then, systematic random sampling was used to select the study participant from each discipline. Hence, 299 participants from Dessie referral hospital (153 Nurses, 20 Midwives, 50 physicians, 19 laboratory technologists, 41 druggist and pharmacists and 16 other health care professionals) and 123 participants from Buru Meda primary hospital(42 Nurses, 11 Midwives, 10 physicians, 8 laboratory technologists, 26 druggist and pharmacist and 26 other health care professionals) were eligible to participate in this study.

### Data collection tool and procedure

The Hospital Survey on Patient Safety Culture (HSOPSC) is a pretested standardized structured self-administered questionnaire that was used to collect the quantitative data, which measured patient safety culture [9]. The HSOPSC emphasizes patient safety and error and event reporting [9]. The questionnaire has 42 items grouped into 12 composite measures, or composites [9]. The tool also includes demographic characteristics of the professionals and other factors.

However, the tool is designed for health care professionals so that patient characteristics, safety experiences and perceptions were not included in the study. Likewise, participant’s participation in patient safety programs were not assessed in the study.

Five diploma trained Nurses collected the quantitative data while the principal investigators conducted the interviews collecting the quantitative data. The nurses required 2 days of training that supervised by the principal investigators. The questionnaire was pretested on 21 participants (5% of the sample size) in another Hospital. Regular monitoring was done during the data collection period by 2 supervisors and the principal investigators.

The level of patient safety culture was measured by the participant’s responses on the HSOPSC questionnaire through a Likert scale and the percentages of the positive responses.

To calculate particular safety culture composite, the percent of positive responses on all items included in the composite were averaged. Negatively worded items were reversed when computing percent positive response.

### Operational definitions

**Good patient safety culture**: score of ≥75% hospital Survey on Patient Safety Culture (HSOPSC) questions.

**Poor patient safety culture**: score of <75% hospital Survey on Patient Safety Culture (HSOPSC) questions.

#### Data processing and analysis

Quantitative data was cleaned, coded and entered into Epi-Info 7 and transferred to SPSS version 20.0 for analysis. Descriptive statistics were computed and presented through tables, graphs, and frequencies. Logistic regression analysis was used to identify an association between dependent and independent variables. Crude and adjusted odds ratios together with their corresponding 95% CI were computed. All predictors that were associated with outcome variable in bivariate analyses with P value < 0.25 was selected to fit for the logistic regression model in multivariate analysis. A P value of <0.05 was declared statistically significant in the multivariate analysis.

### Ethical considerations

Ethical approval and clearance was obtained from the ethics and research review committee of College of Health Sciences, Debre Markos University. Supportive letter was submitted to Dessie referral hospital and Boru Meda primary hospital and permission to conduct the study was obtained from respective hospital’s chief executive officers. A written informed consent was obtained from each study participant after explaining the objective and rational of the study. All the data collected from participants was recorded anonymously and confidentiality was assured throughout the study.

## Results

### Sociodemographic characteristics

Four hundred and eleven participants completed the questionnaire providing a response rate of 97.4% where 422 was the calculated sample size. More than half of the participants, 232(56.4%) were male. The mean age of the participants was 29 years +/-3.85. Nearly two thirds of participants, 59.4% (n = 244) self-identified as Orthodox Christians. The majority of the respondents (87.3%) had less than ten years of work experience (Table 1).

**Table 1:** Socio-demographic characteristics of health care professionals at public hospitals in Dessie town, Northeast Ethiopia, April 2019 (N = 411).

**Variables**		**Frequency(N)**	**Percent (%)**
Sex	Male	232	56.4
	Female	179	43.6
Age (years)	<25	57	13.9
	25–34	331	80.5
	≥34	23	5.6
Religion	Orthodox	244	59.4
	Muslim	147	35.8
	Others	20	4.9
Profession	Physicians	56	13.6
	Nurses	190	46.2
	Midwives	31	7.5
	Pharmacist	67	16.3
	Laboratory tech	27	6.6
	Others	40	9.7
Educational level	Diploma	80	19.5
	Degree and above	331	80.5
Work experience in years	<10	359	87.3
	≥10	52	12.7
Marital status	Single	247	60.1
	Married	144	35
	Others	20	4.9
Monthly salary(in birr)	<3000	42	10.2
	3000–6000	234	56.9
	>6000	135	32.8

### Facility and work-related characteristics

One hundred and four (25.3%) of respondents worked on the gynecology and obstetrics ward. More than 90% (n = 379 (92.2%)) of the respondents had not received training related to patient safety. Nearly two-third (n = 279(67.9%)) of respondents reported they were not satisfied with their job (Table 2).

**Table 2:** Facility and work-related characteristics of study participants at public hospitals in Dessie town, Northeast Ethiopia, April 2019 (N = 411).

**Variables**		**Frequency(N)**	**Percent (%)**
Hospital type	Referral	292	71
	Primary/district	119	29
Working unit/departments	Medical and Surgical	103	25.1
	Pediatrics	46	11.2
	Gynecology/Obstetrics	104	25.3
	Psychiatry	13	3.2
	ICU	20	4.9
	OR	30	7.3
	Emergency	33	8.0
	*Others	62	15.1
Perceived job satisfaction	Satisfied	132	32.1
	Not satisfied	279	67.9
Training related to patient safety	Yes	32	7.8
	No	379	92.2
Shifting type	Every 8 hours	321	78.1
	Regular(day)	90	21.9
Direct contact with patients	Yes	365	88.8
	No	46	11.2

*Others: Radiology, Ophthalmic, Physiotherapy, Dental, ART, TB/MDR-TB, OPD, MCH

### Level of patient safety culture dimensions

Less than half (n = 184 (44.8%)) with a 95% CI of 40.4–49.4 perceived good patient safety culture. Teamwork with in hospital units (74.14%), teams working across hospital departments (53.14%) and the supervisor’s expectation (51.94%) were the highest positively contributing dimensions for overall patient safety culture (Table 3).

**Table 3:** Patient safety culture dimensions at public hospitals in Dessie town, northeast, Ethiopia in 2019 (N = 411).

No	Patient safety culture dimensions	Number of Items	Positive safety culture score (%)
1	Team work with in hospital units	4	74.14
2	Team across hospital department	4	53.14
3	Supervisor expectation and action promoting safety	4	51.94
4	Overall perception of patient safety	4	51.24
5	Organizational learning	3	40.24
6	Communication openness	3	42.74
7	Hospital management support for patient safety	3	33.94
8	Hospital handoffs and transition	4	42.24
9	Staffing	4	40.54
10	Feedback and communication about error	3	47.34
11	Frequency of event reporting	3	34.64
12	Non-punitive response to error	3	25.44
	**Overall level of patient safety culture**	**42**	**44.8**

### Factors associated with patient safety culture

In the bivariate analysis, type of profession, level of education, work experience, age, hospital type and working units were associated with a patient safety culture.

However, in multivariate analysis only age, hospital type and working units were significantly associated with patient safety culture.

Health care professionals in ages between 25–34 years were 75% (AOR = 0.25, 95% CI = 0.08–0.74) less likely to have good patient safety culture compared to those who were ≥34 years.

Health care professionals working in primary/district hospital were three times more likely (AOR = 2.56, 95% CI = 1.56, 4.21) to have good patient safety culture than health care professionals working in the referral hospital.

Health care professionals working in Pediatrics and Emergency ward/unit were 61% (AOR = 0.39, 95% CI = 0.17–0.9), and 75% (AOR = O.25, 95%CI = 0.09–0.67) less likely to have good patient safety culture compared to those who were working in medical and surgical ward (Table 4).

**Table 4:** Association between health care professional characteristics and patient safety culture at public hospitals in Dessie town, Northeast Ethiopia, 2019 (N = 411).

Variables	Category	Patient safety culture(PSC)		COR (95% CI)	AOR (95% CI)	P value
		Good	Poor			
Age	<25	34	23	0.64(0.23–1.81)	0.46(0.13–1.59)	0.22
	25–34	134	197	0.29(0.11–0.74)	0.25(0.08–0.74)	**0.01**
	≥34	16	7	1.00	1.00	-
Hospital Type	Referral	112	180	1.00	1.00	-
	Primary	72	47	2.46(1.59–3.81)	2.56(1.56–4.21)	**0.0001**
Profession	Physicians	16	40	1.00	1.00	-
	Nurses	95	95	2.5(1.31–4.76)	3.88(0.41–36.47)	0.23
	Midwives	11	20	1.37(0.53–3.5)	3.44(0.30–38.99)	0.31
	Pharmacists	32	35	2.28(1.3–7.15)	5.02(0.50–50.19)	0.16
	Laboratory technicians	8	19	1.05(0.38–2.88)	1.51(0.13–16.48)	0.73
	**Others	22	18	3.05(1.07–4.85)	3.35(0.33–33.16)	0.30
Level of Education	Diploma	46	34	1.89(1.15–3.10)	3.91(0.29–39.89)	0.26
	Degree and above	138	193	1.0	1.00	-
Working unit	Medical and Surgical	51	52	1.00	1.00	-
	Pediatrics	11	35	0.32(0.14–0.69)	0.39(0.17–0.9)	**0.02**
	Gynecology/Obstetrics	63	41	0.64(0.33–1.22)	0.6(0.25–1.41)	0.24
	Psychiatry	6	7	0.87(0.27–2.77)	0.95(0.25–3.5)	0.94
	ICU	6	14	0.43(0.15–1.22)	0.42(0.13–1.33)	0.14
	OR	15	15	1.02(0.45–2.29)	0.99(0.4–2.4)	0.98
	Emergency	8	25	0.32(0.13–0.79)	0.25(0.09–0.67)	**0.006**
	Others	24	38	0.64(0.33–1.22)	1.63(0.83–0.3.19)	0.14
Work experience in years	<10	149	210	1.00	1.00	-
	≥10	35	17	2.9(1.56–5.37)	1.48(0.69–3.18)	0.30

1 = Reference

* = P–value <0.05 (significant).

**Others: Physiotherapy, Radiology, Dental, Ophthalmic, Anesthesia, ART, TB/MDR-TB, Chronic OPD.

## Discussion

In this study, the overall level of good patient safety culture was (44.8%). The result is in line with previous studies conducted in Jimma (46.7%), Amhara region public hospitals (46%) and South Africa (42.4%) [8,10,11]. However, the finding was lower than studies done in International Hospital Survey on Patient Safety (HSOPS)(68.8%), Iranian hospitals (50.1%) and Sri Lanka (81.3%) respectively [12–14]. Likewise, the result was higher than studies conducted in Egypt and Taiwan which was (40.2%) and (36%) respectively [15,16]. This difference might from differences in institutions structure, type of professionals and educational level.

Among the twelve dimensions of patient safety culture, team work within hospital units, teamwork across hospital departments and supervisor expectations were the highest contributing dimensions for overall good patient safety culture. The result is congruent with a study conducted in Amhara region [11]. On the other hand, in this study hospital management support for patient safety (33.94%) and non-punitive response to error (25.44%) were the least contributing dimensions of patient safety culture while a study conducted in Sri Lanka showed workload and staff (15.7%) and frequency of events reporting as it occurs (36.3%) were the least contributing dimensions [14].

This study revealed that health care professionals in ages between 25–34 years were 75% less likely to have good patient safety culture compared to those who are ≥34 years. This finding is similar with studies, done in Riyadh, in Northern China and in Kuwait [17–19]. The possible explanation for this could be as age increase; experience, social interaction, attitudes, perceptions, and values that staffs share within an organization related to patient safety increases.

Health workers working in primary hospital were three times more likely to have good patient safety culture than health care professionals who worked in a referral hospital. The result is in line with a study done in Bhutan, in Ethiopia and in Lebanon [11,20,21]. The possible reasons could be in referral hospitals; there is increased patient flow with sever diagnosis and multiple health care needs. This might result in overloaded health care professionals and decreased perception of patient safety culture.

Health care professionals who were working in Pediatrics and Emergency ward/unit were 61% and 75% less likely to have good patient safety culture compared to those who were working in medical and surgical ward. The result is similar with previous study done in Sweden [22]. The similarities might be related to patient characteristics, case type and tasks. Working in Pediatric and Emergency ward requires relatively special skills and commitments. Therefore, it has significant burden on the health care professional’s patient safety culture.

In this study, type of profession, level of education and work experience were not associated with good patient safety culture. The result is contrary to the study conducted in Ethiopia [11] where nurses reported better in the overall patient safety score compared with other health care professionals and in other studies where decreased trend prevailed for overall perception of safety as work experience increases [21] and increased perception of patient safety culture as professionals had more work experience [23].

## Limitation of the study

As all the data were collected through self-administered questionnaire, a real observation of a practice was not done.

We didn’t include patient’s experiences that could bring new knowledge to the result.

## Conclusions

The overall patient safety culture in this study was under 50%. Team work across hospital, team work within units and supervisor expectations were the highest contributing dimensions for overall good patient safety culture. Participant’s age, hospital type and working ward were significantly associated with patient safety culture. Developing patient safety guideline and implementing actions that support all dimensions of safety culture should be promoted at all levels of hospitals for all professionals. Also, hospital leaders and managers should facilitate and support staff rotation among different wards.

## Supporting information

S1 File (SAV)
